# Advancing the Selection of Neurodevelopmental Measures in Epidemiological Studies of Environmental Chemical Exposure and Health Effects

**DOI:** 10.3390/ijerph7010229

**Published:** 2010-01-19

**Authors:** Eric Youngstrom, Judy S. LaKind, Lauren Kenworthy, Paul H. Lipkin, Michael Goodman, Katherine Squibb, Donald R. Mattison, Bruno J. Anthony, Laura Gutermuth Anthony

**Affiliations:** 1 Departments of Psychology and Psychiatry, University of North Carolina at Chapel Hill, Davie Hall, CB 3270, University of North Carolina, Chapel Hill, NC 27599, USA; E-Mail: Eric.Youngstrom@unc.edu; 2 LaKind Associates, LLC, 106 Oakdale Avenue, Catonsville, MD 21228, USA; 3 Department of Epidemiology and Preventive Medicine, University of Maryland School of Medicine, Baltimore, MD 21202, USA; 4 Department of Pediatrics, Penn State College of Medicine, Hershey, PA 17033, USA; 5 Children’s National Medical Center, Center for Autism Spectrum Disorders, Departments of Pediatrics, Neurology and Psychiatry, George Washington University School of Medicine, 15245 Shady Grove Road, Suite 350, Rockville, MD 20850, USA; E-Mails: lkenwort@cnmc.org (L.K.); LAnthony@cnmc.org (L.G.A.); 6 Center for Development and Learning, The Kennedy Krieger Institute, Department of Pediatrics, The Johns Hopkins University School of Medicine, 707 North Broadway Baltimore, MD 21205, USA; E-Mail: lipkin@kennedykrieger.org; 7 Department of Epidemiology, Emory University School of Public Health, 1518 Clifton Rd., Atlanta, GA 30322, USA; E-Mail: mgoodm2@sph.emory.edu; 8 Department of Medicine, University of Maryland School of Medicine, 11 South Paca Street, 2nd Floor Baltimore, MD 21201, USA; E-Mail: Ksquibb@umaryland.edu; 9 Eunice Kennedy Shriver National Institute of Child Health and Human Development National Institutes of Health, Department of Health and Human Services, Building 31, Room 1B44, Bethesda MD 20892-7510, USA; E-Mail: mattisod@mail.nih.gov; 10 Georgetown University Center for Child and Human Development, Department of Pediatrics, 3300 Whitehaven Street, NW, #3300, Washington, DC 20007, USA; E-Mail: bja28@georgetown.edu

**Keywords:** neurodevelopmental measures, neurodevelopment, polychlorinated biphenyls, PCBs, children’s health, domain, psychometrics, developmental epidemiology

## Abstract

With research suggesting increasing incidence of pediatric neurodevelopmental disorders, questions regarding etiology continue to be raised. Neurodevelopmental function tests have been used in epidemiology studies to evaluate relationships between environmental chemical exposures and neurodevelopmental deficits. Limitations of currently used tests and difficulties with their interpretation have been described, but a comprehensive critical examination of tests commonly used in studies of environmental chemicals and pediatric neurodevelopmental disorders has not been conducted. We provide here a listing and critical evaluation of commonly used neurodevelopmental tests in studies exploring effects from chemical exposures and recommend measures that are not often used, but should be considered. We also discuss important considerations in selecting appropriate tests and provide a case study by reviewing the literature on polychlorinated biphenyls.

## 1. Introduction

Many underlying causes for childhood neurodevelopmental disorders have been explored, including early (e.g., fetal, perinatal) exposures to environmental chemicals [1]. Methods for assessing adverse effects on neurodevelopment are broadening to include fetal neuroimaging (including functional magnetic resonance imaging, or fMRI), and toxicogenomics. Nevertheless, in environmental epidemiology studies, neurodevelopmental function tests form the basis for evaluations of associations between chemical exposure and human health effects.

The uses of neurodevelopmental tests in studies of environmental chemicals and pediatric neurodevelopmental disorders have been reviewed [2–4] and limitations of currently used tests and the difficulties with their interpretation have been described [5,6], for example in relation to long-term consistency of test outcomes. However, a comprehensive critical examination of commonly used tests in environmental epidemiology has not been conducted. In addition, many commonly used measures in other research areas (e.g., neuropsychology) have not gained wide use in the environmental chemical study arena and deserve attention.

In this paper, we seek to advance the science of neurodevelopmental function testing in environmental epidemiology studies by identifying central issues that should inform the choice of assessment devices for inclusion in future studies. These include general issues such as the relative merits of measures that capture broad versus narrow neurodevelopmental processes or domains (*i.e.*, the function/neurodevelopmental process being assessed; for example, IQ is a broad cognitive measure, processing speed is a narrow cognitive measure), as well as technical concerns that arise when attempting to use new measurement strategies while maintaining connections with prior literature. We also make recommendations about guiding principles that can facilitate the design of neurodevelopmental studies, as well as specific suggestions about choices of measures and domains to provide a prototype—not a rigid template—for successful future investigations. Specifically, the following are reviewed: (i) commonly used neurodevelopmental measures (*i.e.*, test or instrument) and measures that are not often used, but should be considered, by environmental epidemiologists, (ii) methodological issues that influence study findings, and (iii) methods for measuring other risk and protective factors that impact findings.

Although most environmental chemicals have not undergone extensive evaluations for their effects on neurodevelopment, a few chemicals (e.g., lead, methylmercury, polychlorinated biphenyls [PCBs]) have been studied by multiple research groups over many years. We selected PCBs as a case study for critically reviewing commonly used neurodevelopmental tests in environmental epidemiology studies because it offered a sufficient number of studies to provide a meaningful basis for evaluation of assessment methodology without requiring review of a prohibitive number of articles. We do not discuss specific outcomes reported in the individual studies, nor do we weigh in on the potential merits or weaknesses of past studies. Rather, we use the list of neurodevelopmental function tests employed in assessments of childhood neurodevelopment and PCBs as the foundation for a discussion of key aspects of test selection that must be considered when designing these types of studies. We then give recommendations for a path forward that might strengthen the use of these tests to support risk assessment. It is hoped that this exercise will serve as the foundation for multi-disciplinary discussions regarding best practices in the field of neurodevelopmental environmental epidemiology. A template for best practices is essential as these epidemiological studies (in conjunction with toxicological studies) form the foundation for risk assessment and regulation of many environmental chemicals.

## 2. Experimental Section

Our strategy was to identify key primary and review articles for a selected chemical class and review them to build an initial list of measures and domains [7,8]. We used PCBs as our chemical class as several epidemiological studies of neurodevelopment have been conducted and have included a wide range of measures [9]. We then searched for updates, revisions, and competing versions of those measures. We identified “incumbents” or the measures most frequently used across studies; the most frequently used in each domain are evaluated in Tables 1 and 2. For each measure, two independent raters (LA, LK) nominated an additional measure that would improve upon the incumbent. When the raters disagreed (which happened for three of the measures), the evidence base related to the measures was discussed and a consensus reached.

The measures most commonly used in epidemiological studies of PCBs are shown in Table 1. For the purposes of this research, each version of a measure was treated as a discrete entity and each distinct component of each measure was evaluated as a distinct entity. In reviewing the measures, we noted the domain labels assigned by the test developer, by the epidemiological investigators, by reviewers of the literature (e.g., [9]) and also according to current practice in neuropsychology. When the labels for domains were inconsistent, we organized Tables 1 and 2 around current practice, rather than historical or study-specific assignments.

## 3. Results and Discussion

The primary goal of this study was to identify and evaluate measures that have been commonly used in epidemiological studies examining environmental chemical effects on neurocognitive development. For new research projects that are not designed solely for hypothesis-generation to be compelling, they need to build on prior research by including additive, incremental advances and newer components that reflect current advances in theory and technique. A project’s neurodevelopmental assessment battery (typically comprised of several measures) must be broad enough to capture relevant domains, but focused enough to be feasible. The measures themselves need to balance developmental appropriateness against the competing virtue of maintaining comparability across a wide age range. Additionally, measures have different strengths and weaknesses in terms of their psychometric properties (*i.e.*, reliability, validity, population samples upon which the measure is normed). Viewed through the lens of designing an optimal neurodevelopmental study, not all psychometric features are equally important.

Measures used in epidemiological studies of PCBs and alternative measures suggested for future studies are shown in Table 1. Most of the PCBs studies used versions of tests that were current at the time of the study, but the majority of the commercially-distributed measures have been updated since the completion of the cohort studies under review here. Table 2, which is designed to serve as a resource for environmental epidemiologists, gives detailed information on various properties of neurodevelopmental measures. Together, Tables 1 and 2 should provide sufficient information for researchers to select the best neurodevelopmental measures that cover their domain of interest. We hope the comprehensive list will also inspire researchers to use different tests than those used in previous studies, thus building upon past studies by including more sensitive measures or new areas of interest.

During our review of the test batteries used in prior research and of subsequent developments with measures, we identified a set of cross-cutting themes and methodological issues pertinent to the design of new studies as well as the evaluation of published studies; these are described in following subsections. Examples from Tables 1 and 2 are used to highlight these issues. As is clear from these tables, a large number of measures have been used to assess potential effects of PCBs (which is presumably only a subset of a much larger list if additional toxicants are considered). The complete set of tests included in Table 2 is too large for any single cohort study to include or for future studies to fully incorporate. Reasonable principles or guidelines are needed to help investigators select measures that connect with prior research and also take advantage of any improved assessment tools; we provide recommendations on this topic as well.

The review of the PCB literature and associated neurodevelopmental tests, as well as the exploration of alternative recommended tests, brought to light several important methodological issues to consider when designing a study and choosing assessment measures. Each issue is outlined below, followed by recommendations for future environmental epidemiology research.

### 3.1. Neurodevelopmental Measures and Domains

Evaluations of results of neurodevelopmental studies as part of a weight-of-evidence assessment (the process used in hazard evaluation to evaluate the degree of certainty regarding the adverse health effects of a chemical) necessarily include a review of the domains studied. This evaluative process, crucial to risk assessment, would be aided by consistent interpretations regarding the domain that a measure examines. However, the review of the PCBs literature revealed variation in the ways that neurodevelopment was parsed into domains, and also variations in how tests were categorized as measures of particular domains. A further complication is that different fields of study do not always use the same domain definitions, making interdisciplinary communication difficult (e.g., see differences in how domains are categorized in Table 1 versus categorization used by Boucher *et al.* [9]). This is not surprising, as it reflects the evolution of domain definitions that do not have distinct boundaries. The fact that many tasks have multiple components or involve coordination between multiple systems of functioning adds to the challenge. For example, the Arithmetic subtest from the Wechsler versions of the intelligence tests for children and adolescents asks the subject to listen to a story problem and then perform arithmetic operations in their head before producing an answer. As a result, the task includes auditory processing (listening to the passage), verbal processing (identifying the quantities and operations required), working memory components (maintaining the key elements in working memory and performing operations on them), an achievement component (having been exposed to and learning the necessary arithmetic operations), plus the nonverbal general ability component that would be expected based on the content and the subtest name (Sattler, 2001). Because of the task complexity, the Arithmetic subtest has been found to statistically relate more to the Verbal IQ and the Freedom from Distractibility Composite Index, but never significantly to the Nonverbal IQ or Perceptual Organization Composite Index (or later analogs). This illustrates the point that tests can be difficult to categorize even using quantitative and objective methods, let alone rational or theory-driven models.

Recommendations: It is clear that there have been changes over time and across studies in how assessment tests are categorized. A consistent rubric should be developed and adopted, even though it would necessarily be imperfect, provisional, and subject to periodic revision.

### 3.2. Broad versus Narrow Measures

Most of the neurodevelopmental studies of PCBs used a combination of broad and narrow measures. “Broad” in the neurocognitive sense refers to measures that use composite scores to summarize performance across multiple tasks, with the composite score acting as an indicator of a complex underlying domain. Examples include the composite index scores or full scale summary score from intelligence tests. “Narrow” refers to measures that assess a more focal process or construct. Examples include visual-motor, articulation, or spelling abilities.

**Figure 1.**
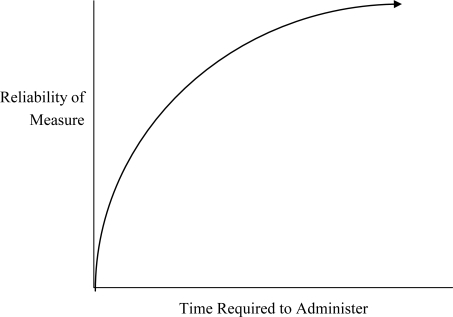
Relationship between the length of a measure and its reliability.

Broad and narrow measures both have advantages and disadvantages. In general, the reliability, validity, and predictive value of a measure increase with the length of the test [44] (Figure 1). An advantage of broad measures (e.g., IQ) is that they are typically measured with greater reliability because they integrate information from multiple components, resulting in a longer test less influenced by error affecting any one component.

This is a fact of psychometrics: The longer and more thorough the test, the more precise the estimate of the “true score”—the person’s level of the ability or trait, uncontaminated by error or other factors not related to the construct of interest. A second advantage of broad measures is that they tend to be based on factor analysis, which provides the important conceptual advantage that measurement is organized around the underlying domain of interest, not just observed performance on a test. Scores on a vocabulary test, for instance, can be influenced by educational opportunity, personality factors, language development, and a variety of other factors in addition to intelligence; whereas a verbal composite index focuses on the underlying ability that is shared across a vocabulary test as well as analogies, measures of general knowledge, and other tasks. Broad measures are thus more reliable and potentially more “pure” measures of some domains. A third major advantage of broad measures is that they have the greatest predictive value in terms of relating to educational, occupational, and health outcomes. General cognitive ability has consistently proven to be one of the most robust predictors of functional and vocational attainment [45,46] and has a surprisingly powerful association with health, longevity, and other important outcomes [47]. A fourth potential advantage is that more broad, global measures of performance may be sensitive to the cumulative effects of multiple decrements across a set of underlying, more focal processes (as a hypothetical example, a chemical could negatively impact working memory and processing speed; the broader measure could capture the confluence of these impacts, which more closely mirrors what one would observe in the child’s everyday life).

The disadvantages of broad measures are in many ways the converse of the strengths. Estimating a broad score requires that the test sample from a variety of different domains, creating pressure for longer test length and greater expense and burden. Within the cognitive ability literature, the tension between the competing aims of precise estimation of global abilities versus minimizing burden has been partially solved in two ways: choosing the most important subtests and choosing the most predictive items. An approach to shortening battery length without compromising the estimate of overall cognitive ability is to concentrate on an abbreviated battery that includes only the tasks most correlated with the underlying factor. This is the method guiding the use of two-subtest brief batteries (typically a vocabulary and a matrix or block design task), and it also is the rationale for the development of several four subtest measures of ability (*i.e.*, designed and validated specifically as four subtest instruments) (e.g., Wide Range Intelligence Test, or WRIT; [48]; and the Wechsler Abbreviated Scales of Intelligence, or WASI; [22]). A second, more technical approach is to use a family of statistical methods known as “item response theory” (IRT) to guide the selection of test items so that the tests provide the most precise estimate of ability possible with the minimal number of items [49]. IRT methods have been incorporated into the selection of items for the instruments designed to be brief batteries (e.g., WASI and WRIT). IRT methods also can be used in an “adaptive testing” framework, where computer administration makes it possible to select subsequent items based on individual performance on earlier items. Adaptive testing makes it possible to achieve equally precise estimates with roughly 30% fewer items administered, but it requires computer administration. Adaptive testing will be become increasingly feasible to add to epidemiological studies as computer administration of other performance tests becomes more commonplace.

The advantages of narrow measures (e.g., Beery Test of Visual-Motor Integration) include greater brevity and a more direct connection to a specific neurocognitive process or brain region. There is also the potential for narrow measures to be more sensitive to neurotoxic effects on specific systems or areas of the brain [4]. However, detection of effects on narrow tasks is made harder by the lower reliability and sometimes unknown but often lower validity of task performance as a measure of an underlying domain. A major issue is that performance on a single task can be influenced by multiple variables. Sattler [50], for example, lists between nine and two dozen variables that can affect performance on each of the subtests comprising a Wechsler intelligence test. When multiple subtests are available, it is possible to use techniques like factor analysis to uncover the underlying domains of interest; but with an individual test it is not possible to disentangle the potential sources of error and variation. Some tasks, such as the Wisconsin Card Sorting Test (see Table 1) [17], are now recognized to be intrinsically complex and involve multiple neurocognitive processes for the person taking the test. At the same time, some narrow measures relate to an underlying function or domain that may truly stand alone.

In the educational assessment literature, there has been much discussion of “cross battery assessment” as a means of improving the measurement of specific domains. The main concept in cross-battery assessment relies on choosing several different tests that are supposed to measure the same domain, though often drawn from different published tests. For example, to provide good measurement of working memory, the three subtests from the WISC-IV might be supplemented with two more tests from the Wide Range Assessment of Memory and Learning. There are a variety of technical obstacles to the implementation of this cross-battery assessment strategy, some of which would be tractable in a large-group epidemiological study because it would be possible to redo factor analyses on the measures in question within the epidemiological study [51].

Recommendations: Given the largely complementary strengths and weaknesses of broad versus narrow measures, an optimal strategy for future environmental epidemiology studies would be to include a mix of both broad and narrow measures. Broad measures are best at estimating real world functioning and provide the most reliable and valid measurement options. Narrow measures are still important, however, because they may identify specific neurocognitive impacts that may not be observed with the broad measures. The choice of narrow measures should be tailored to each study based on prior evidence and specific hypotheses or questions about neurodevelopmental vulnerabilities potentially linked to the toxicant. However, studies that include a large number of narrow tasks without *a priori* motivation based on the literature or theory will create more problems than they solve. Increasing the number of batteries incurs costs of greater expense, increased burden, more missing data, inflated Type I errors or false positive results, less parsimony and more potential redundancy in findings. There is also the potential for Type II or false negative errors if psychometrically weak measures fail to detect true neurodevelopmental effects.

It is possible to use newer, brief, well-validated measures to provide precise estimates of global functioning. For example, using a four subtest battery provides equally precise estimates of general cognitive ability and verbal or nonverbal functioning as would be obtained using a corresponding ten or twelve subtest battery. The choices of narrow tests should be informed in part by prior research, making sure to include domains that previously have been found to be affected by exposures to toxicants. The battery can also be supplemented by some narrow measures chosen for conceptual reasons.

### 3.3. Old versus New Versions of a Measure

An important issue is the basis for choosing between using newer versus older versions of measures. The ethical guidelines of the American Psychological Association and other professional organizations clearly state that practitioners should use the most current version available for each measure [52]. The most appropriate measure for a practitioner may differ from that of a researcher. However, benefits for the researcher using the current version of a measure include: (a) enhanced generalizability of findings from the research cohort into clinical practice—at least until the measure in question is updated again; (b) congruence with ethical guidelines for practice; (c) gaining any theoretical or psychometric advantages built into the revision of the measure; and (d) avoidance of problems due to differences in the older standardization sample versus the population to which the investigator or others wish to generalize results.

However, there are costs associated with adopting newer versions of measures, especially in the context of conducting repeated assessments on a cohort of interest. If a cohort completed a particular version of a measure at study inception, then it would simplify the research design to continue administering the same version of the measure at follow-up periods (ignoring the constraints of practice effects—the effect associated with improvement on a test simply due to repeated administration—or developmental appropriateness). Using the Wechsler Intelligence Scales for Children (WISC) as an illustrative example, if at the start of the study the WISC-III (Wechsler Intelligence Scales for Children, 3^rd^ edition) was used, but the WISC-IV is the current version available, then it is not a simple matter to switch to the new version of the measure and compare the scores. Each revision from WISC to WISC-R to WISC-III and WISC-IV has involved the addition or the subtraction of subtests. Each revision has changed the underlying factor structure of the battery [50], with some subtests (e.g., Arithmetic) migrating from one composite index into a different composite index. As a result, comparisons of two composite scores with the same name (e.g., Verbal IQ) are complicated by the fact that they might not be based on the same underlying set of tasks, and newer batteries may omit composite scores that were included on previous versions of the measure (e.g., the WISC-IV no longer provides Verbal IQ and Performance IQ estimates). Adding to the complexity are changes in names for composite scores, which are usually intended to reflect theoretical models or reconceptualizations, but nonetheless add to the challenge of describing results (as when “Freedom from Distractibility” changes into “Working Memory”, sometimes with an additional subtest added to the composite score).

There are other issues involved in changing versions of measures. One is the change in standardization samples. Most measures are interpreted by comparing the raw score to the average score for peers of the same age or demography (*i.e.*, the standardization sample). Standardized scores are created by comparing individual performance to the standardization sample. The methods for constructing the standardization sample vary widely, from local convenience samples of cases in a single clinic or community to stratified samples that are designed to be nationally representative. At present, the best normative samples typically are available for intelligence tests and measures that are co-normed in the same sample with them. However, these samples typically involve aggregating many smaller convenience samples distributed throughout the country of interest (Table 2 includes scored evaluations of the type and quality of the standardization samples in the measures used in PCBs studies, revealing a full range from small clinical convenience samples to population-level studies).

When conducting studies on effects of toxicants, researchers selecting a battery need to be cognizant of the composition of the standardization sample and how it compares to the sample included in their study. The discrepancy between the standardization versus participant samples causes problems when the norms are based on a US sample and the participants come from other countries (e.g., differences in language, culture). An obvious example is on the WPPSI test, which includes a picture of a child kicking an American football; the test requires accurate identification of this activity as “football” to earn full credit; this would be an unfair question to most of the rest of the world.

Discrepancies can also be meaningful within the same country. A standardization sample that was matched to national demography in 1970 will under-represent Latino Americans if the study sample was collected in 2009. Similarly, a test with nationally representative norms based on the year 2009 could still under-represent Latino Americans if the sample gathered for the environmental epidemiology study was drawn primarily from a heavily Latino region such as Texas. All of the cohorts studied in the PCBs literature were drawn from relatively geographically circumscribed regions, not from stratified nationally representative samples. This suggests that for epidemiological studies of toxicants the more common practice will be to gather samples from subsets of the population. Researchers should carefully consider whether the sample of participants differs from the demography included in the standardization sample. If there are differences, then the researchers should review the literature to determine whether these factors are associated with differences in performance on the neurodevelopmental test in question. If so, then the analytic plan of the study needs to address the potential confounding variables, at a minimum by including the potential confounders as correlates. Failure to do so could result in the appearance of seeming deficits that actually are due to cultural or demographic factors, and not due to the environmental exposure. These differences need not be limited to effects of culture or language on measures of academic knowledge or intelligence [44]; there also will be regional differences in diet or prevalence of genes that may be associated with performance on more narrow measures as well as potentially conferring differences in susceptibility to environmental exposures. For example, there are sizeable epidemiological differences in the distribution of the DRD4 alleles that are associated with sensation-seeking and impulsivity [53], and it is likely that there will be other differences in distribution of genes that influence performance on narrow measures.

Another potential confound related to changes in standardization samples is the possibility of temporal trends that alter the performance of the sample on the tasks. The most critical example of this is the “Flynn Effect,” where performance on tests of general cognitive ability has been found to increase by an average of roughly three points per decade [54]. This pattern has been observed across multiple measures and multiple samples from different countries around the world. Thus it appears to be a general trend, although there is no clear explanation for why performance would be improving globally [55]. For the purposes of an epidemiological researcher, the practical consequence is that observed scores will appear lower on newer versions of tests (because the scores are being compared to the new, higher average level of performance). If a study is conducted such that a cohort first gets an older version of a measure, such as a WISC-III, and then the cohort is followed up with a WISC-IV, scores might be expected to drop 3 to 5 points at the later assessment due to the change in the norms, and not due to any actual change in performance. It would be a mistake to attribute this effect to long-term sequelae of the environmental exposure. Although the Flynn Effect represents a small effect size, this could generate spuriously large differences in the percentage of cases with extreme scores (see section on Clinical Significance below). Unfortunately, there is no easy solution to the confound introduced by the change in norms. For example, analyzing the raw scores would not be workable because (a) average performance changes rapidly with age—hence the need for age-based norms; (b) the actual item content of the subtests will change between versions; (c) sometimes entire subtests change between re-standardizations of the battery. If there is a linking sample of cases that took both the old and the new versions of the test (which is often done as part of the updating process for new versions of measures), then it may be possible to estimate the size of the Flynn effect and the extent to which it might influence performance on particular measures.

Recommendations: Researchers will almost always want to use the newest available versions of measures at the beginning of a study. They will want to become familiar with the differences between the new version and older versions that may have been used in prior published studies. Differences in subtest composition, factor structure, and constitution of the standardization sample all become confounding variables and would rival hypotheses for any differences in patterns of findings. If repeated assessments are performed on the same cohort, then consideration needs to be given to the benefits of using consistent measures versus switching to newer tests when an older version might still be viable. If the primary purpose is within-subjects comparisons looking at trajectories over time within the cohort, then a good case would be made for retaining the older test even though a different version becomes available. Some of the technical issues with changes in version and norms will be unavoidable when the cohort ages across the boundaries between different versions of tests, such as the transition from preschool to school-aged, or adolescence to adulthood. Interestingly, many of the brief four-subtest versions of intelligence measures have broader age norms (e.g., 6 to 80 years versus 6 to 16 years), and they also may be less prone to changes in subtest content or factor structure than the larger batteries. These attributes may make them attractive candidates for many epidemiological studies. Researchers should also bear in mind that these factors affect comparisons between samples more than they affect correlations within the same sample: Using a particular version of a test may provide an accurate estimate of the association between toxicant exposure and neurocognitive functioning, even though the test may provide biased estimates of average functioning compared to the normative sample.

### 3.4. Psychometrics: Conventional and Relevant Metrics

Test publishers provide information about the psychometric properties of instruments, including various measures of reliability (referring to the reproducibility of scores) and validity (referring to evidence that the instrument actually measures what it is designed to measure) [44,56,57]. It is crucial for investigations into environmental impacts on neurodevelopment to include consideration of the psychometric properties of the measures selected when designing the study. There are many different ways of measuring both reliability and validity. Information on these issues is discussed in the following subsections and included in Table 2.

#### Reliability

One form of reliability is internal consistency, indicating the extent to which different parts of a test are measuring the same domain. Internal consistency is the single most widely reported measure of reliability, due to the fact that it is the least expensive type of reliability data to gather, not because it is intrinsically superior to other forms of reliability. For the purposes of epidemiological studies of toxicants, internal consistency often may be the least relevant of the major forms of reliability coefficients in guiding the selection of measures. It is also possible for internal consistency to be “too high” in some circumstances. Most indices of internal consistency are influenced by scale length, such that longer scales tend to be more internally consistent. Two items with very similar content will also correlate more highly than two items measuring different aspects of the same domain. For example, responses to items asking whether the participant “feels down” and “feels blue” would show greater internal consistency than would “feels down” and “insomnia,” even though all three items are relevant to the domain of depression. As a result, concentrating on maximizing internal consistency may paradoxically result in selecting scales that are longer than necessary, and may favor more redundancy or narrowness of domain representation rather than broad coverage with less internal consistency [57].

Another form of reliability is inter-rater reliability, which refers to the extent to which scores are reproducible when the same test is administered or scored by different individuals (“raters”) administering the measure [57]. Many tests involve scoring decisions, including judging the quality of verbal responses and assigning them to zero, one or two-point categories on vocabulary tests, or timing the speed at which block patterns are duplicated and making decisions about what constitutes an acceptable degree of rotation in the orientation of the pattern. These decisions introduce opportunities for human error and also for a degree of subjectivity in the decision-making; thus, it is important to evaluate the degree of reproducibility of scores across raters [50]. This issue also applies to giving neurological assessments, reading x-ray or MRI images, and many other classification decisions [58]. Cicchetti *et al.* [59] provide a review of different benchmarks for describing inter-rater reliability and some thoughts about selection of measures in terms of trade-off between reliability and validity.

Retest stability refers to the extent to which individuals tend to maintain the same scores upon repeated administrations, such that high scorers on the initial assessment also tend to be the highest scorers when taking the test again. Retest stability is usually indexed as a correlation between the two sets of scores, thus ignoring overall changes in the level of scores. Retest stability tends to diminish as a function of time between administrations, such that two-week stabilities would be higher than two-year stabilities. Stability also varies as a function of the domain being assessed. As per the state versus trait distinction in psychology, some individual differences are expected to vary substantially across time and situation (state variables, e.g., sleep deprivation), whereas others are expected to show greater temporal and situational stability (trait variable, e.g., IQ). Stability also increases with age. For instance, the two-year stability of performance on a cognitive variable is likely to be much greater in the period between 24 and 26 years of age than would be found for the same dimension between 4 and 6 years of age.

**Figure 2.**
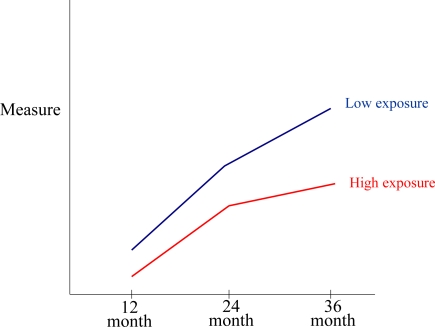
Hypothetical developmental trajectories for low-exposure and high-exposure groups.

In the context of environmental epidemiological studies, retest stability can be informative by suggesting which tasks might be expected to show greater spontaneous recovery (or regression to the mean in the event of low stability) [60]. If the study design includes a low-exposed comparison cohort, then between-group comparisons provide a way of examining change in the effects of exposure over time; and if three or more administrations are available, then we recommend growth-curve modeling techniques as a way of comparing group differences in developmental trajectories (Figure 2) [61,62]. Where available, the basic information on reliability is included in Table 2.

#### Validity

There are several types of psychometric validity. The most important to the environmental epidemiology literature are construct validity, predictive validity, exposure sensitivity, and ecological validity.

Construct validity: Construct validity describes the extent to which a measure satisfies multiple underlying forms of validity (e.g., the extent to which the measure includes appropriate content, correlates with other established measures of the same domain, correlates with measures of different but related domains, and discriminates among diagnostic groups) [44,63]. Where available, the basic information on construct validity is included in Table 2.

Predictive validity: Predictive validity refers to concurrent or prospective predictions and was used by Davidson *et al.* [4] to evaluate tests for environmental epidemiology studies. The value of longitudinal prediction in a neurodevelopmental framework is clear. Concurrent predictive validity can also be called diagnostic efficiency when the measure is demonstrating validity in terms of assigning children into categories such as clinical diagnosis. Diagnostic efficiency is most commonly reported in terms of sensitivity and specificity, where sensitivity refers to the percentage of children that truly have the target condition who are classified correctly, and specificity refers to the rate of children who do not have the target condition who are classified correctly [64]. A challenge in using diagnostic efficiency is that there needs to be a gold standard indicator of “true” status against which the assessment tools can be evaluated. For environmental epidemiological studies, the choice of criterion diagnoses could include definitions such as presence/absence of mental retardation, presence/absence of clinically significant impairment, or other definitions. For diagnostic efficiency statistics to be readily interpretable, the criterion needs to be dichotomous. However, this raises important questions about whether taking a criterion that could be measured continuously (such as cognitive ability) and converting it to a category (such as mental retardation versus within normal limits) loses information and reduces statistical power to detect effects [65]. There are considerable communication and policy advantages to using a dichotomous definition [66]; however, it must be recognized that important information is lost in this process, especially in terms of clinical significance. Adopting the framework of diagnostic efficiency would also provide methods for dividing individuals into the dichotomous groups based on costs and benefits attached to correct identification and avoidance of errors [66–68]. Some groups have already used the diagnostic efficiency framework to evaluate the performance of candidate tests at discriminating between known groups, such as low birth weight versus normal birth weight, or learning disabled versus not [4]. This approach is an approximation, in that known categories (low birth weight, learning disability) are being substituted for an unknown category (effect of toxicant), and the specific effects of a toxicant may be different from the signature effects of low birth weight. However, the results demonstrated that the majority of the assessments investigated could not discriminate to a statistically significantly degree between known groups, raising serious concerns about their assay sensitivity if used in epidemiological studies. Where available, the basic information on the predictive validity of measures used in PCBs neurodevelopmental research is included in Table 2.

Exposure sensitivity: This is similar to the concept of “treatment sensitivity” in the clinical trials literature: Has a measure demonstrated an ability to pick up the signal of a treatment effect when there is other evidence that the effect is present? This type of validity information is almost completely absent from the technical manuals or primary publications describing the psychometric properties of tests reviewed for this research. There were some exceptions, including the Bayley-III (see Tables 1 and 2) technical manual’s presentation of scores for children who were exposed to alcohol *in utero* per mother report, resulting in small effect sizes for decrements in gross and fine motor ability (*d* ~ 0.3), moderate deficits in cognitive ability (*d* ~ 0.6) and large deficits on language ability and socio-emotional functioning (*d* ~ 0.8). The same manual also provided information about average scores for a sample of infants that suffered asphyxia at birth (again per maternal report), with associated average deficits in the moderate range across all scales (*d* = 0.3 to 0.7) [18].

The advantages of the “exposure sensitivity” approach are that the statistical methods will be familiar to the scientific community, and it is often easier to assign people to groups based upon exposure status instead of outcome status (although there has also been concern about the heterogeneity and imprecision of definitions of exposure in the literature) [69]. Demonstration of sensitivity to exposure effects offers evidence that a measure can overcome the problems of imperfect reliability and validity to detect a measurable outcome. Even when found, exposure effects need to be interpreted with caution, for example studies that use a large number of tests or statistical comparisons increase the risk of false discovery (meaning detecting a statistically significant result by chance; this risk can be reduced by using a false detection rate correction to the p value to determine significance). Prior success at detecting exposure effects provides a method for streamlining batteries by eliminating instruments that have failed to detect effects, and also concentrates more attention and resources on tools that detect larger effects.

Ecological validity: Ecological validity is the ability for a measure to relate to real world functioning [63,70]. Many past environmental epidemiology studies have not included measures that focus specifically on everyday functioning. In Tables 1 and 2, we include measures that have been shown to have improved ecological validity. For example, epidemiology studies have used continuous performance tasks (CPT, a computerized test of attention). However, research in the field of ADHD shows that parent and teacher rating scales are better at detecting clinically significant differences in attention functioning. We therefore recommended the Conners Rating Scale if the goal is to identify meaningful behavioral effects, whereas the CPT might be a better “narrow” measure of attention processes (Table 1).

Recommendations: For the purposes of detecting the effects of toxicant exposure, conventional psychometric properties will not be equally important. Nor does the frequency with which psychometric characteristics are reported align with the degree of importance for epidemiological studies. Internal consistency is probably less useful for appraising candidate tests than inter-rater reliability or retest stability, but internal consistency is far more commonly reported in the primary publications and technical manuals of the assessment tools reviewed (Table 2). Using computer-assisted testing increases the standardization of administration and scoring for complex tasks, reducing a source of inter-rater reliability error and potentially enhancing the power of research designs to detect exposure effects (e.g., see Table 2, the CTONI, WCST or CPT). For applied purposes, higher inter-rater reliability is always desirable; but when comparing measures it is important to recognize that different designs can produce different reliability estimates. Inter-rater reliability will generally be much higher when judges are given the same audiotape or transcript to rate versus conducting separate interviews with the participant (adding variability due to administration as well as variability in scoring). We recommend evaluating the psychometric properties of each measure used with the study’s sample, when possible, and comparing those properties to those found in the standardization sample. It is probably most important to evaluate inter-rater reliability in the test administrators/scorers regularly during the course of a study. We recommend growth-curve modeling techniques as a way of comparing group differences in developmental trajectories

Similarly, predictive validity and exposure sensitivity are two highly relevant but rarely reported parameters. We recommend increased emphasis on reporting the relevant parameters, both in technical manuals and in research reports, to facilitate improving test selection. We also recommend a multi-tiered approach to test selection, where tests that have demonstrated exposure sensitivity may be supplemented by a second tier of other tests chosen on a theoretical basis, and perhaps a third tier of exploratory measures if resources permit.

### 3.5. Cultural Effects

Cultural effects are a major consideration in test selection. Most tests only have a standardization sample and normative data available in one language, even if the instrument has been translated into multiple languages. Translation is a complex process, and even with fluent translators and blinded “back-translation” into the original language for review, there can be important cultural differences in the way concepts are expressed. There can also be differences in the behaviors of interest on which the measure focuses. For example, there might be differences in the way that cultures experience depression. There might also be culture-dependent differences in the relationship between an item asking if the person “cries a lot” and their underlying level of depression. In addition, there may be differences in the amount of crying that is typical in a culture, independent of the underlying level of depression. These issues can be formally investigated using both qualitative techniques (ethnographic interviews and focus groups) as well as quantitative methods. However, with regard to neurodevelopmental tasks, most of the research about cultural effects is in its infancy.

The current shortcomings of research on cultural effects leave limited options for environmental epidemiologists. If the battery is constructed to avoid verbal or culturally loaded tasks, then the range of measures is constrained, and many of the tests with the strongest relationships to functional outcomes or behavior would be excluded. If only tests with thorough cultural adaptation and separate norms are used, then only a few instruments are added to the available pool. Reliance on tools that have been translated but not validated introduces potential confounds that should at a minimum be acknowledged as a potential limitation. Ideally, if the sample size is large enough and analytic resources are available, then examining the stability of the psychometrics using multi-group statistical methods would become a valuable secondary aim for the research [71].

Recommendations: We recommend increased resources be dedicated to research on cultural effects. Few of the tests we reviewed have been translated, and even fewer have normative data available for the translated version. We recommend that researchers use measures with similar levels of translation and validation, report them accurately in the measures sections of papers, and discuss the potential limitations in their reports. When selecting measures, it will be important to include some tests that have minimal verbal components. We do not recommend avoiding verbal tests, though, particularly if a goal of the investigation is to generalize to functioning in everyday settings. A secondary aim of projects with adequate resources would be to use qualitative and statistical methods to evaluate the degree of measurement equivalence when tests are transported into different languages and cultures.

### 3.6. Measuring Other Risk and Protective Factors

Most of the environmental epidemiological studies under review recognized the importance of measuring other factors besides toxicant exposure that could affect the individual’s outcome. In addition to measuring comprehensive demographics (place of residence, parental age, race, marital status, *etc*.), medical status of the child and mother during pregnancy and birth, birth order of the child measured, age at exposure, severity of exposure, exposure to other important toxicants (e.g., smoking in the home, prenatal alcohol exposure, lead) and route of exposure, there are several other important factors that could either increase or decrease the severity of the effects. For example, nutrition has been measured in some studies and found to act as an important moderator [72]. Breastfeeding has also been shown to act as a protective factor. Socio-economic status (family income, parent education and parent occupation) is known to have profound effects on neurocognitive development and should be measured in every study. Studies have also used the Home Observation for Measurement of the Environment (HOME; [73]) to measure quality of home environment in a standardized manner as it is also known to have profound effects on development. Parental verbal ability/IQ is often reported as a covariate, though the most commonly used measure (Peabody Picture Vocabulary Test, or PPVT) [74] is not a culture-free test and should, therefore, be used with caution. Additionally, a child’s overall cognitive ability acts as a protective factor regardless of the endpoint of interest. Such influential factors as cognitive ability should be included statistically as covariates.

Recommendations: It would be useful for investigators from multiple disciplines to pre-determine a set of variables that should be considered as covariates for every study, and suggest a systematic way of measuring those variables to increase the ability to make direct comparisons among studies and cohorts. For example, when measuring socioeconomic status, some investigators in the studies we reviewed used the Hollingshead Scale [75], some used education and income separately, and others created a unique approach using combined percentiles. A consistent method that could be used cross-culturally would be preferable. The demographic variables that are routinely described as features for standardization samples should typically be included as covariates, especially if the group exposed to the toxicant might differ on any of these features from the comparison group. If the research design includes different levels of exposure to the toxicant (e.g., exposed versus unexposed, unexposed versus single exposure versus multiple exposure, or more commonly, different amounts of exposure), then including interaction terms between the covariate and the exposure variable in the statistical approach will markedly reduce bias in the estimates of effects for the toxicant [76]. Another struggle relates to balancing the importance of measuring the possible covariates with the time required to measure some of them well. If investigators are looking for a more culture-free but still quick estimate of parental IQ, they might consider a measure such as the Test of Nonverbal Intelligence, 3^rd^ Edition, (TONI-3 [77]), which is similar to the Ravens Progressive Matrices [78], but with much more recent norms.

### 3.7. Statistical Significance versus Clinical Significance

A recurring theme in the clinical literature is the distinction between statistical significance versus clinical significance; this issue has also been raised in the context of environmental epidemiological studies [79]. This distinction has proven challenging to use in practice, but it is also highly relevant to discussions of measuring the effects of toxicants on neurodevelopment.

Statistical significance most commonly refers to situations where the observed results (*i.e.*, the study’s findings) fall outside of a confidence interval (range of scores) around the result that would have been expected under a null hypothesis (*i.e.*, a finding of no difference between groups); or similarly, when a test statistic evaluating an observed finding exceeds a critical value for the desired level of significance. In a study of toxicants, a statistically significant result would mean that the differences between the exposed and unexposed groups (or high exposure versus low exposure groups) were large enough that they would only have been observed by chance “rarely”—with “rarely” typically being defined as less than 5% of the time. Sometimes results are presented as an estimate of the effect size of exposure (see below) with a confidence interval that indicates the upper and lower bounds of the estimate. If the confidence interval is set at 95%, then this is conceptually equivalent to testing against a null hypothesis with an alpha <0.05.

Statistical significance thus establishes a crucial filter for evaluating the potential effects of toxicants. Significant results indicate that the toxicant has an effect on the measure, or else might be a false positive result (*i.e.*, a result obtained by chance alone; if a study makes 20 comparisons, one of those comparisons, or 5%, might be a “rare” difference observed by chance alone). Nonsignificant results indicate that the toxicant is weak or inert with regard to that particular measure, or else that the study might have produced a false negative error (*i.e.*, there is a true difference that could not be detected by the study because of other factors such as poor measurement, poor inter-rater reliability, cultural effects, or not enough children in the study to detect a result). For the purposes of toxicant research, false positives are costly: They can lead to unnecessary increases in concern about exposure, and perhaps unnecessary regulatory action, management and/or treatment. False negatives are at least equally worrisome, as they can lead to the erroneous conclusion that the compound is safe—at least in regard to that particular measure – thus perhaps resulting in less regulation and potentially greater exposure. Using psychometrically weak measures increases the risk of failing to detect effects that are actually present (false negatives). Running a large number of significance tests on a battery containing multiple measures increases the risk of false positive results. Both errors should ideally be avoided, but research study design balances them against each other.

Methods for increasing statistical power (*i.e.*, the ability to detect a “true” difference) in environmental epidemiological studies include: (1) using a more liberal definition of significance (*i.e.*, adopting a more lenient alpha level), (2) increasing the size of the effect, and (3) decreasing the size of the error in estimating the effect. The first option, using more liberal definitions of significance, directly increases the risk of false positive errors. The other options, increasing the effect size and reducing error are methods that can increase power without inflating the risk of false positives, so they are clearly preferable.

Methods for increasing the size of the effect include increasing the exposure level (in human studies, this would translate into identifying and including subjects known to be highly exposed) and focusing on the neurocognitive areas that are maximally affected by the exposure. Increasing exposure may be acceptable in animal models but raises obvious ethical issues in human models. Thus, the most effective approach for increasing power in studies of toxicants is reduction of error.

Techniques for reducing error include increasing the size of the sample, increasing the precision of the measurement of effects (e.g., choosing one’s measures wisely as outlined above), eliminating variance due to extraneous sources (e.g., confounders and covariates), and using repeated measures designs (ideally combining pre-exposure and post-exposure measurements on the same individuals). Pre-post designs are again often difficult to conduct with humans and toxicants, as ethical values will dictate relying on accidental exposure and other “natural experiments” which make it difficult to collect pre-exposure levels (though the large, prospective National Children’s Study may contain pre-post components [80]). However, the other two approaches appear promising as ways of increasing statistical power in many studies of toxicants. Adopting measures with better psychometric properties will improve measurement precision, thus reducing error and improving power. The alternative measures in Table 1 are recommended because of their strong psychometric properties.

Statistical significance is a necessary but not sufficient condition for evaluating the effects of a toxicant. If a toxicant effect does not achieve statistical significance in well-designed studies with adequate statistical power, and especially if it remains nonsignificant across multiple studies, then the interpretation would be that the toxicant does not have a meaningful effect on that outcome measure. On the other hand, it is possible to achieve statistical significance with effects that are too small to be clinically meaningful or to have policy implications (e.g., attaining statistical significance with small effects if they are measured with great accuracy or with large samples). A readily-understood example of this is as follows: Measured with enough precision, most people have one foot that is longer than the other (leading to a correct rejection of the null hypothesis of equal foot length); but the difference is rarely large enough to justify buying a different sized shoe for each foot (requiring a change in shoe purchasing policy). Conversely, there are examples where a even a small effect should result in a response (e.g., reducing heart attack risk with preventive treatment with low-dose aspirin).

The precision of a measure suggests a natural benchmark for comparison of observed effects. “Accuracy” typically is reported as the standard error of the measure, or the precision with which observed scores estimate the true score. If IQ tests are typically accurate to +/− 3 points, and change scores on IQ tests are only accurate to +/− 4.5 points at the individual level, then effect sizes that are smaller than 3–4 points are not impressive considering the precision of the tool. Although the standard errors are rarely reported in articles (they are more common in technical manuals), they can be estimated based on the standard deviation and the reliability of the instrument (see Table 2 for information on measures’ standard errors). When the same test is given more than once, the precision of the difference between the two scores is lowered by the imprecision in both the first and second testing. This “standard error of the difference” is always 41% larger (the square root of two) than the standard error of the measure.

Another way to assess clinical significance is by comparison with benchmarks established by normative data for the measure. The most important benchmark is located two standard deviations away from the average score for the standardization (or in the case of toxicants, the unexposed) sample. This definition establishes a meaningful and consistent threshold that could be applied with any test that has normative data. However, this would capture only the most extreme or frank effects. This method sets a much higher threshold compared to using the standard error of the measure and the difference between groups.

The use of a consistent definition of clinical significance would be valuable, as many test manuals and interpretive systems advocate for the use of more idiosyncratic thresholds (e.g., [37]), and investigators also adopt different definitions across studies.

Recommendations: Statistical significance testing provides a first filter to separate effects that will probably be reproducible from those that are so small that any observed effects could be attributed to sampling variation rather than exposure to a toxicant. The chance of detecting an effect when it is present in the population—statistical power—can be enhanced in several ways. However, many of the conventional methods for improving power are problematic for epidemiological studies of toxicants. Methods that could be used to further enhance power include using factor analysis or covariance structure modeling to better assess underlying domains and remove the effects of measurement error, or inclusion of covariates chosen because they can control for variance in the outcome measures that is not dependent on exposure to the toxicant.

Statistical significance in and of itself is not necessarily equated with clinical or policy significance. Interpretation of findings from studies of toxicants would benefit from adopting some of the reporting techniques developed in the clinical significance or evidence-based medicine literatures. However, not all of the concepts and techniques will be conceptually relevant, and some will often not be feasible given the practical constraints of doing large-scale studies of exposure to toxicants in humans.

### 3.8. Developmental Effects on Neurocognitive Functioning and Consequent Changes in Assessment Stability and Validity

Developmental brain changes can influence the domain of functioning tested by different instruments, and development also affects the stability and predictive validity of test scores. Brain functioning is less differentiated, and expression is less specific at an early age. As speech, abstract abilities, and meta-cognitive processes develop, different brain regions and processes are recruited in the performance of tasks. These developmental changes imply some instability of outcome with increasing age, especially at younger ages. Thus instability of outcome does not automatically imply that the measurement at early age has been invalid, especially with regard to evaluating contemporary functioning. At the same time, the lower predictive validity associated with measures administered at young ages could be attributable to resilience or to difficulty assessing the construct at a younger age (e.g., it may be impossible to evaluate impaired reading ability in a preverbal child).

## 4. Conclusions

We reviewed the measures used to assess neurodevelopmental effects of toxicants, concentrating on those measures previously used in the PCBs epidemiology literature. We found that:

- there are a large number of measures that have been used, including both global and more narrowly-focused measures;
- there have been continued revisions and changes to many of the core measures, which necessitate changes in the selection of tests for new research protocols;
- entirely new measures are available that warrant consideration for inclusion in new studies of toxicants due to their superior psychometric properties;
- entirely new domains should be explored in new studies of toxicants due to their importance in real world functioning and/or the possibility that they would be sensitive to toxicants’ effects (e.g., adaptive functioning, executive functioning, articulation);
- the most commonly documented psychometric properties for measures (such as internal consistency reliability estimates or concurrent validity correlations) are only indirectly relevant to the main objectives of epidemiological studies of toxicants;
- the most relevant psychometric features for measures used in toxicant studies (such as retest stability or sensitivity to exposure effects) have been reported only rarely;
- the selection of covariates in environmental studies has been largely focused on demographics and confounders, whereas the inclusion of other covariates (e.g., IQ) that are highly correlated with the dependent variable (e.g., language) would further improve estimation of the effects of toxicants;
- the field of environmental epidemiology may be nearing a stage where a formal set of reporting guidelines could be developed to help the design of future studies, as has been done with clinical trials, studies of diagnostic assessment tools, and medical epidemiological studies;
- in terms of domains, it is clear that there have been changes over time and across studies in how assessment measures are categorized. A consistent rubric should be developed and adopted, even though it would necessarily be imperfect, provisional, and subject to periodic revision;
- predictive validity and exposure sensitivity are two highly relevant but rarely reported parameters. We recommend increased emphasis on reporting the relevant parameters, both in technical manuals and in research reports, to facilitate improving measure selection.

We also recommend a multi-tiered approach to measure selection, where measures that have demonstrated exposure sensitivity may be supplemented by a second tier of other measures chosen on a theoretical basis, and perhaps a third tier of exploratory measures if resources permit. Our comprehensive list of measures will be used by researchers to build upon past studies by including more sensitive measures or new areas of interest.

## Figures and Tables

**Table 1.** Examples of tests used in PCB epidemiology literature and alternative recommended measure(s) for each domain. There were three possible bases for the recommended alternative measure: (1) the recommended measure has more advantages and fewer disadvantages (as enumerated in Table 2), (2) the recommended measure addresses an important domain that had been unexplored in past studies, or (3) the recommended version is a newer measure with updated norms.

**Measure**	**Exists in PCBs literature [E]/Recommended alternative for future studies [R]**	**Scale Name**	**Rationale for recommended alternative measure (see above)**
*Academic Achievement*			
Wide Range Achievement Test	E (WRAT 3^rd^ Edition)/R (WRAT 4^th^ Edition)	Word Reading Sentence Comprehension Reading Composite Spelling Math Computation	3
Woodcock-Johnson-III	R	Academic Fluency Subtests	1
*Adaptive Behavior*			
Adaptive Behavior Assessment System-II	R	Parent Form Global Assessment of Competence	2
Vineland Adaptive Behavior Scale-II	R	Parent Interview Edition	2
*Attention*			
Conners’ Continuous Performance Test (CPT II)	E	Sustained attention Omissions d Prime Commissions Variability Standard Error	NA
Conners Rating Scales, Third Edition	R	Conners III Total Score	1
ADHD Rating Scale	R	Inattention Hyperactivity/Impulsivity	1
*Executive Function—Omnibus*			
Wisconsin Card Sorting Test (WCST)	E	Multiple scores	NA
Behavior Rating Inventory of Executive Functioning (BRIEF)	R	Global Executive Composite	1
*Executive Function—Flexibility*			
Wisconsin Card Sorting Test (WCST)	E	Perseverative Errors	NA
BRIEF	R	Flexibility Index	1
*Executive Function—Organization/Planning*			
Rey Complex Figure Test	E	Copy Strategy	NA
Tower of London-DX	R	Total Move Score	1
*Executive Function—Response Inhibition*			
CPT II	E	Commissions	NA
BRIEF	R	Inhibit Scale	1
*Executive Functioning—Working Memory*			
Wechsler Intelligence Scale for Children-Revised (WISC-R)	E	Arithmetic	NA
Wechsler Intelligence Scale for Children, 4^th^ Edition (WISC-IV)	R	Working Memory Index	3
*General Cognitive Measures:Infants and Toddlers*			
Mullen Scales of Early Learning	E	Early Learning Composite	NA
Bayley Scales of Infant Development	E/R (3^rd^Edition)	Adaptive behavior Cognitive Language Composite Motor Composite	3
*General Cognitive Measures: Preschool and Older*			
McCarthy Scales of Children’s Ability (MSCA)	E	General Cognitive Index (GCI) Verbal Perceptual-Performance Memory	NA
Differential Abilities Scale-II (DAS-II)	R	General Cognitive Ability Verbal Ability Nonverbal Ability Spatial Ability	1
*General Cognitive Measures: Childhood and Older*			
Wechsler Intelligence Scales for Children—Fourth Edition (WISC-IV)	E (WISC-R)/R (WISC-IV)	Full Scale Verbal Comprehension Perceptual Reasoning Working Memory Processing Speed	3
Wechsler Adult Intelligence Scales (WAIS-III)	E (WAIS-R)-R (WAIS-III)	Full Scale Verbal Performance Verbal Comprehension Perceptual Organization Working Memory Processing Speed	3
Wechsler Abbreviated Scale of Intelligence (WASI)	R	Full Scale Verbal Performance	1
*General Cognitive Measures: Non-verbal*			
Comprehensive Test of Nonverbal Intelligence (CTONI)	R	Nonverbal Intelligence Composite Pictorial Nonverbal Intelligence Composite Geometric Nonverbal Intelligence Composite	2
Leiter	R	Visualization & Reasoning Attention & Memory	2
*Gross, Fine Motor Function*			
McCarthy Scales of Children’s Ability	E	Motor	NA
Peabody Developmental Motor Scales	R	Fine Motor Quotient Gross Motor Quotient	1
Finger tapping	R	Finger tapping raw scores	1
*Language—Expressive Language*			
Verbal subtests from IQ measures (e.g., WISC, MSCA)	E	Vocabulary, Information, Similarities, Comprehension	NA
Clinical Evaluation of Language Fundamentals (4^th^ Ed.) (CELF)	R	Expressive Language	1
Pre-School Language Scale (PLS 4)	R	Auditory Comprehension Expressive Communication	1
*Language—Receptive Language*			
CELF	R	Receptive Language	2
PLS 4	R	Auditory Comprehension Expressive Communication	2
*Language—Articulation*			
Goldman-Fristoe Test of Articulation	R	Sounds in Words Sounds in Sentences Stimulability	2
*Language—Pragmatic Language*			
Test of Problem Solving—Child and Adolescent (TOPS)	R	Pragmatic Language	2
*Learning/Memory-Verbal*			
California Verbal Learning Test-II (CVLT-II)	E (CLVT-II)/R (CLVT-II, 2^nd^ Edition)	Total Correct	3
*Learning/Memory-Visual*			
Wide Range Assessment of Memory and Learning, 2^nd^ Edition (WRAML-II)	R	Visual Memory Index	2
*Maladaptive Behavior*			
Achenbach Child Behavior Checklist (CBCL)	R	Total Problems Externalizing Internalizing Attention Problems	2
Aberrant Behavior Checklist (ABC)	R	Irritability; Lethargy; Stereotypy; Hyperactivity; Inappropriate Speech	2
Infant-Toddler Social and Emotional Assessment (ITSEA)	R	Problem Total; Competence Total; also Externalizing, Internalizing, Dysregulation, Competence, and Maladaptive	1
*Processing Speed*			
CPT II	E	Reaction time (Conner's)	NA
WISC-IV	R	Symbol Search subtest	1
*Social Cognition*			
Social Responsiveness Scale (SRS)	R	SRS Total Total - Parents (Female) Total - Parents (Male) Total - Teachers (Male) Total - Teachers (Female) Clinical Ratings (Both)	2
*Visual Motor*			
Beery Test of Visual Motor Integration, 5th Ed. (VMI)	E	Visual Motor Total Score	NA
*Visual Spatial*			
WASI	R	Performance IQ	2

**Table 2.** Description (including advantages and disadvantages) of widely used neurodevelopmental measures and alternate recommended measures (see Table 1). Norm quality was rated on a four point scale: ****=Exemplary, with nationally representative demographics and good sample size across relevant age spans, *** = Good, with some shortcomings (such as dated norms, coarsely clustered sampling, or omission of important group), ** = Suboptimal (e.g., badly out of date, or convenience sample that was not nationally representative), * = Flawed.

**Measure**	**Scale Name**	**Age Range** (yrs unless otherwise indicated)	**Admin. Time**	**Norm (N)/Norm Quality**	**Reliability (Type)**	**Standard Error of Measurement**	**Stability (r)**	**Construct Validity**	**Predictive Validity**	**Advantages**	**Disadvantages**	**References**
				**Standard Score M (SD)**								
*Academic Achievement*												
Wide Range Achievement Test 4	Word Reading Sentence Comprehension Reading Composite Spelling Math Computation	5– 94 yr 11 mo	15–25 minutes for ages 5 to 7 for whole test; 30–45 minutes for over age 7 for whole test	3021/**** _______ 100 (15)	0.96 (median alpha); 0.90 immediate retest alt. form 0.96 (median alpha); 0.86 immediate retest alt. form 0.98 (median alpha) 0.95 (median alpha); 0.89 immediate retest alt. form 0.94 (median alpha); 0.88 immediate retest alt. form	3.0 3.0 2.3 3.4 3.7	0.85 *r* for alternate form delayed test retest (*Mean*=1 month; range 8 to 86 days) 0.74 *r* for alternate form delayed test retest (*M*=1 month; range 8 to 86 days) 0.88 *r* for alternate form delayed test retest (*M*=1 month; range 8 to 86 days) 0.83 *r* for alternate form delayed test retest (*M*=1 month; range 8 to 86 days) 0.83 *r* for alternate form delayed test retest (*M*=1 month; range 8 to 86 days)	Good: Moderate to high correlations with other achievement measures	Some evidence of predictive validity in terms of educational classification	Short, alternative forms allows re-testing, part can be administered in group format	Captures basic learning difficulties with reading decoding, and math computation, but is not sensitive to learning disabilities associated with executive function, processing speed, motor output, reading comprehension, or written expression.	[10]
Woodcock-Johnson-III	Academic Fluency Subtests	2 to 90+	Variable, ~5 min. per test	8818/**** 100 (15)						Relatively easy to administer; sensitive to the effects of processing speed and motor output deficits on academics.	Moderately old norms	[11]
*Adaptive Behavior*												
Adaptive Behavior Assessment System-II	Parent Form Global Assessment of Competence	Birth to adult	15–20 min	1350/**** 100 (15)	0.97 (alpha)	2.12	0.88 (2 days to 5 weeks, *M*=12 days)	Extensive	Used in identification of mental retardation	Multiple versions for different ages and parents and day care providers; extensive construct validity	Like any parent checklist, ABAS is susceptible to misinterpretation and bias.	[12]
Vineland Adaptive Behavior Scale-II (a brief research edition is also available)	Parent Interview Edition Parent Form Global Assessment of Competence (GAC)	0–18 5–21	20–60 minutes and 15–30 minutes to score 15–20 min	1670/**** 1670/**** 100(15)	0.98 (alpha)	1.57	0.93 (5 days to 6 weeks; avg of 11 days)	Extensive	Used in identification of mental retardation	Well validated in multiple clinical groups Self-report version; multiple versions for different ages and parents and day care providers; extensive construct validity	Time and expertise intensive measure for the interview version; can take more than 1 hour to complete. Administration of interview version requires expertise gained through graduate level training programs in psychology or social work.	[13]
	Teacher Form GAC Teacher Form GAC	2 to 5 5 to 21	15–20 min 15–20 min	750/**** 100 (15) 1690/**** 100 (15)	0.98 (alpha) 0.99 (alpha)	2.94 1.97	0.91 (2 days to 6 weeks, avg of 13 days) 0.96 (3 days to 3 weeks; avg of 11 days)	Extensive Extensive				
*Attention*												
Conners, 3^rd^ Edition	Conners III Total (also a short form, a DSM form, and a global form)	6 to 18	5–20 min	1200 parents, 1200 teachers, 1000 youths/*** 50 (10)	0.91 parent, 0.94 teacher, 0.88 youth (alpha)	1.7 to 4.8, depending on scale	0.85 parent, 0.85 teacher, 0.79 youth (2–4 week interval)	Extensive	Discriminates ADHD from normal or clinical comparisons; sensitive to treatment effects in multiple trials	Parent, teacher, and youth forms (no Global Index on youth version); includes DSM-IV content; extensive research base; includes validity scales	Cumbersome to score without computer software; short forms validated in embedded version (not separate administration)	[14]
CPT II	Sustained attention Omissions d Prime Commissions Variability Standard Error	6+ (A pre-school version is also available)	15–20 min	1920^C^/*** 50 (10)	0.87 (split half)	Range of SEM is: 2.6 to 4.6	0.65 (Average retest interval of 3 months, N only 23)	Moderate	Less predictive than behavior scales	Standardized task that measures multiple performance facets of attention	Relatively small number of minorities included in the norm sample; overall mild correlations between CPT and ADHD rating scales	[15]
*Executive Function—Omnibus*												
Behavior Rating Inventory of Executive Functioning (BRIEF)	Global Executive Composite	2 to adult	10–15 min	1419/** 50 (10)	0.98 (alpha, parent and teacher)	1.41	0.81 parent 3 week; 0.91 teacher 3.5 week	Good	Some evidence of predictive validity for diagnoses	Parent and teacher forms; inexpensive; collateral source of information about executive functioning. Comprehensive coverage of subdomains of executive functioning; ecologically valid measure; used extensively in research with good sensitivity; easy to administer and complete.	Parent rating are susceptible to bias; report of everyday executive function does not necessarily accurately parse subdomains of executive function. Normative sample not nationally representative; variable correlations between scores and underlying processes	[16]
*Flexibility*												
Wisconsin Card Sorting Test (WCST)	Perseverative Errors	6.5 to 89 yr 11 mo	20–30 min	5 samples^A^/*** 100 (15)	0.92–0.97 for perseverative errors (inter-scorer, ICC)	10.39 for perseverative errors in child/adolescent; 11.91 for % perseverative errors in child/adolescent	0.52 for 1 mo test-retest perseverative errors; 0.37 for 1 mo test-retest (n=46) for percent perseverative errors	Moderate -- group differences	None	Relevant construct for neurotoxicity	Difficult to reliably score if not using computer administration; not representative norms; complex relationship between scales and executive function	[17]
*General Cognitive Measures: Infants and Toddlers*												
Bayley Scales of Infant Development	Adaptive behavior Cognitive Language Composite Motor Composite	1 to 42 months	50–90 minutes	1700/**** 100 (15)	0.99 (split half) 0.91 (split half) 0.93 (split half) 0.92 (split half)	3.11 0.95 4.47 4.42	0.92 0.81 0.87 0.83	Moderate to good (0.6 for similar scales)		One of the only instruments available in the age range, recently re-standardized, extended floors and ceilings, improved evidence of reliability and validity	Difficult to administer; and confounded by significant language demands.	[18]
Mullen Scales of Early Learning (AGS Edition)	Early Learning Composite (Also five subscores: Gross Motor; Visual Reception; Fine Motor; Receptive Language; Expressive Language)	Birth to 68 months	~15 min (for 1 year olds) to 60 min (for 5 year olds)	1849/*** 100 (15) [50 (10) for the five subscores]	0.91 (split half)	4.5	0.71 to 0.96 (median = 0.84) (1 to 2 week interval)	Factor validity; good convergent validity with Bayley	Discriminates low birth weight from normal; predicts school readiness on Metropolitan test longitudinally (two years later)	Limited language demands	Old normative data	[19]
*General Cognitive Measures: Childhood and Older*												
Wechsler Intelligence Scales for Children – Fourth Edition (WISC-IV)	Full Scale Verbal Comprehension Perceptual Reasoning Working Memory Processing Speed	6 to 16	60–90 min	2200/**** 100 (15)	0.97 (split half) 0.94 (split half) 0.92 (split half) 0.92 (split half) 0.88 (split half)	2.68 3.78 4.15 4.27 5.21	0.89 (~1 month) 0.89 (~1 month) 0.85 (~1 month) 0.85 (~1 month) 0.79 (~1 month)	Excellent Excellent Good Good Good	FSIQ - Excellent prediction of achievement criteria; well established use in classification; much less known about factor indices (newer)	Most widely used test of cognitive ability in children and adolescents; excellent norms; familiar; stronger measurement of working memory than previous	Not tied to strong theory of intelligence; relatively weak assessment of processing speed	[20]
Wechsler Adult Intelligence Scales (WAIS-III)	Full Scale Verbal Performance Verbal Comprehension	16 to 89 years	60–90 min	2450/**** 100 (15)	0.98 (split half) 0.97 (split half) 0.94 (split half) 0.96 (split half)	2.12 2.60 3.67 3.00	0.96 (1 month retest) 0.96 (1 month retest) 0.91 (1 month retest) 0.95 (1 month retest)	Exceptional construct validity for broadest scores; stronger construct validity for working memory than in previous versions of WAIS	Extensive	Reliable, norms, more commonly administered and owned (familiar to psychologists)	Not tied to strong theory of intelligence; relatively weak assessment of processing speed and working memory	[21]
	Perceptual Organization Working Memory Processing Speed				0.93 (split half) 0.94 (split half) 0.88 (split half)	3.97 3.67 5.20	0.88 (1 month retest) 0.89 (1 month retest) 0.89 (1 month retest)					
Wechsler Abbreviated Scale of Intelligence (WASI)	Full Scale Verbal Performance	6 to 89	30 min	2245/**** 100 (15)	0.96 (split half) 0.93 (split half) 0.94 (split half)	3.08 3.99 3.75	0.93 ~1 month 0.92 ~1 month 0.88 ~1 month	Exceptional construct validity	Good, based on convergence with WISC and WAIS	Validated as a brief measure of verbal, nonverbal, and general cognitive ability; very precise scores; Matrix Reasoning can be administered nonverbally	No coverage of processing speed, working memory, or other aspects of cognitive ability	[22]
*General Cognitive Measures: Non-verbal*												
Comprehensive Test of Nonverbal Intelligence (CTONI)	Nonverbal Intelligence Composite Pictorial Nonverbal Intelligence Composite Geometric Nonverbal Intelligence Composite	6 to 18 yr 11 mo	40–60 min	2901/**** 1 00 (15 for composites); 10 (3) for subtest	0.97 (alpha) 0.93 (alpha) 0.95 (alpha)	2.6 4.0 3.4	0.92 for Nonverbal IQ for 1 month retest; inter-scorer for the subtests (not composites) range from 0.95 to 0.99 (rating same protocols) 0.87 for test-retest 1 mo 0.91 for test-retest 1 mo	Good criterion validity (0.64 to 0.81 correlation w/FSIQ on WISC-III		Minimizes cultural bias	Less predictive of some aspects of functioning than verbally loaded scales; weaker norms at youngest ages	[23]
Leiter, Revised Edition	Visualization & Reasoning (VR); Attention & Memory (AM)	2 to 21	40 to 90 min	1719 (VR) 763 (AM)/*** 100 (15)	0.75 to 0.90 (median 0.82) (split half)	--	0.83 to 0.92 (but time interval not reported in manual)	Content validity based on examiner ratings of item content; convergent with other IQ tests	Some discriminative validity for cognitive delay, to a lesser degree for ADHD	Covers wide age range; minimal bias across cultures; strong theoretical model guiding revision	Special training may be needed for good standardization; AM subtests not very stable over time	[24]
*General Cognitive Measures: Preschool and Older*												
Differential Abilities Scale-II	General Cognitive Ability Verbal Ability Nonverbal Ability Spatial Ability	2.5–17 yr 11 mo	60 min	3480/**** 100 (15)	.96 (split half) .90 (split half) .89 (split half) .95 (split half)	2.91 4.77 5.15 3.4	.92 (used overall standardization sample) .90 .73 .89	Excellent (0.87 w/WPSSI-III)		Good norms, conceptual model, strong psychometrics	No working memory or processing speed	[25]
McCarthy	General Cognitive Index (GCI) Verbal Perceptual-Performance Memory	2 yr 4 mo to 8 yr 7 mo	60–90 min	1032/*** (well-matched to 1970 Census; excluded exceptional children) 100 (15)	0.93 (split half)	3.97	0.90 for 1 month	Excellent correlations with IQ measures, but can have substantial differences in average scores	Good predictive validity of later school functioning (*r* ~0.5); no diagnostic efficiency reported	Exemplary technical manual; engaging, game-like, non-threatening format; may engage shy and minority children more than other tests	Complex administration and scoring (requiring practice), especially for gross motor Norms are more than 20 years old	[26]
*Gross, Fine Motor Function*												
MSCA	Motor	2 yr 4 mo to 8 yr 7 mo	15 min	1032/*** (well-matched to 1970 Census; excluded exceptional children)	0.69 (split half)	8.35	0.33 for “longer term”	Content valid, but not stable	Low to moderate	Engaging	Can be difficult to administer and score (more so than other MCSA subtests)	[26]
Peabody Developmental Motor Scales	Fine Motor Quotient; Gross Motor Quotient; plus 9 subtest scores	Birth to 72 months	2–3 hours (20–30 min per subtest)	2003/*** 100 (15)	0.96 (split half)	3.0	.93 Fine Motor 0.89 Gross Motor (one week retest)	Good evidence of factor and convergent validity	Unknown; goal of test is to measure treatment effects; but relevant data not included in technical manual	Minimal training needed because of clear instructions and objective scoring; easy to administer	Limited data on children with special needs; kit does not include all materials needed for administration; small objects are a choke hazard and need cleaning if mouthed	[27]
Digital Finger-tapping	Digital Finger Tapping	Various norms; college student for digital version	10 minutes with scoring	80/* Raw score (number of taps)	Not reported	Not reported	Not reported	Fair correlation with other fine motor tasks	Unknown	Easy to administer; electronic counter enhances accuracy	Poor norms; limited psychometric data; primarily suited to research use with comparison groups	[28]
Finger Tapping (Halstead-Reitan)	Finger Tapping	15 to 64	10 minutes with scoring	190/*	Not reported	Not reported	Not reported	Fair correlation with other fine motor tasks	Unknown	Easy to administer; widely recognized test	Small and dated norms	[29]
Finger Tapping (Findeis & Weight Meta-Norms)	Finger Tapping	5 to 14	10 minutes with scoring	1591 dominant; 1558 non-dominant hand/*	Not reported	Not reported	Not reported	Fair correlation with other fine motor tasks	Unknown	Easy to administer	Pools data from 20 different studies to create “norms”	[30]
*Language – Articulation*												
Goldman-Fristoe Test of Articulation, 2^nd^ Edition	Sounds in Words; Sounds in Sentences; Stimulability	2 to 21	15–30 min	2350/**** 100 (15)	0.90 to 0.93 (median inter-rater)	4.0 to 4.7	0.98 (within session)	Moderate: Exper review, but limited construct validation data published	Unknown	Strong standardization sample; good norm-referenced scores	Technical information based on administrations by speech pathologists; unclear how results would vary with less trained raters; use with caution with speakers of non-standard English	[31]
*Language--Expressive Language*												
Pre-School Language Scale, 4^th^ Edition	Auditory Comprehension; Expressive Communication	Birth to 6 yr 11 mo	20–45 min	2400/***	0.81 to 0.97 (split half)	2.6 to 9.2	0.82 to 0.95 (1 week)	Good – Expert review of content; convergent with PLS3 and Denver II, evidence of response process validity	Some discriminative validity for language disorders and autism	New norms; Spanish version available (though less technical data available)	Standardized only in English; no information about how bilingual status influences performance (though ~7% of sample was bilingual); potential for marked variability in administration and scoring means that a high degree of training is needed for consistency	[32]
Clinical Evaluation of Language Fundamentals (4^th^ Ed.) (CELF)	Expressive Language	5–21 (A pre-school version is also available)	30–45 min	2,650/****	0.89 to 0.95 (alpha); 0.88 to 0.99 inter-scorer	--	0.90+ (~16 days)	Good – content, response-process, and factor validity	Good for language disability	Easy to learn; computer-assisted scoring; focuses on specific skills and areas of functioning (versus achievement)	18 subtests if do full battery; low reliability for a few subtests	[33]
WISC-R, MSCA	Vocabulary	Various	Variable	Variable/*** 10 (3)	Generally good	Moderate	Good	Good	Good for achievement criteria	Brief; well-normed; clear scoring	Subtest scores reflect multiple component skills and factors	[26,34]
*Language--Receptive Language*												
Clinical Evaluation of Language Fundamentals (4^th^ Ed.) (CELF)	Receptive Language	5–8, 9–12, 13–21	30–45 min	2,650/****	0.89 to 0.95 (alpha); 0.88 to 0.99 inter-scorer		.90+ (~16 days)	Good – content, response-process, and factor validity	Good for language disability	Easy to learn; computer-assisted scoring; focuses on specific skills and areas of functioning (versus achievement)	18 subtests if do full battery; low reliability for a few subtests	[33]
Verbal subtests from IQ measures (e.g., WISC, MSCA)	Vocabulary, Information, Similarities, Comprehension, *etc*.	Various	Various	Various/*** 10 (3)	Good	Good	Good	Good for crystallized ability	Good for achievement criteria	Well-normed; clear scoring; readily available	Not validated as stand-alone tests; scores on single scale driven by multiple factors (not just receptive language)	[20]
*Learning/Memory-Verbal*												
California Verbal Learning Test (CVLT)		5 to adult	30–50 minutes	920/*** 50 (10); some are 0 (1)	0.85 (split half)	3.83	0.61–0.73 for List A (ages 8, 12 & 16 tables for 28 day median test-retest); 0.37–0.78 for Discriminability (ages 8, 12, & 16 tables for 28 day median test-retest)	Some evidence of factor validity and correlations w/other measures of ability		Widely used test of verbal learning and memory, short, measures recognition and recall		[35]
*Learning/Memory-Visual*												
WRAML-II	Visual Memory Index Verbal Memory Index Attention/Concentration	5 – 84 yr 11 mo	60 minutes for all core subtests	1200/**** 100 (15)	0.89 (median alpha) 0.92 (median alpha) 0.86 (median alpha)	5.0 median 4.2 median 5.6 median	0.67 test-retest 0.85 test-retest 0.68 test-retest	Moderately high convergent validity; good discriminant validity		Wide age range; new norms; stronger factor structure than earlier version	Lengthy administration time; often only specific subtests are used.	[36]
	General Memory Index Screening Memory Index		20 min		0.93 (median alpha) 0.93 (median alpha)	4.0 median 4.0 median	0.81 test-retest 0.78 test-retest (Mean time b/w all tests = 49 days, range 14 to 401 days.					
*Maladaptive Behavior*												
Achenbach Child Behavior Checklist	Total Problems Externalizing Internalizing Attention Problems	1.5 to young adult	10–15 min	1753/**** 50 (10)	0.97 (alpha) 0.94 (alpha) 0.90 (alpha) 0.86 (alpha)	1.73 2.45 3.16 3.74	0.94 ~8 days; 0.81 ~12 mos 0.92 ~8 days; 0.82 ~12 mos 0.91 ~8 days; 0.80 ~12 mos 0.92 ~8 days; 0.70 ~12 mos	Good to excellent	Excellent predictive validity of diagnoses and long term longitudinal outcomes	Multiple versions, multiple informants, forms and norms for multiple age ranges, large research and clinical literature with wide variety of medical conditions	Omits some content likely to be relevant, including theory of mind, mania scale; scales do not map directly onto psychiatric diagnoses.	[37]
Aberrant Behavior Checklist (ABC)	Irritability; Lethargy; Stereotypy; Hyperactivity; Inappropriate Speech	5 to 51+	~5 min for a rater familiar with subject’s behavior	754 New Zealanders; 508 USA (both residential with mental retardation)/**	0.86 to 0.95 (alpha)	Varies across scales and ages	0.96 to 0.99 (4 week retest)	Good factor validity; good convergent validity with other rating scales	Moderate discriminative validity; good treatment sensitivity	Good content coverage; sensitive to treatment effects	Manual provides incomplete psychometric information; much technical data in outside sources; although often used as parent or teacher rating, less validation of these formats	[38]
Infant-Toddler Social and Emotional Assessment (ITSEA)	Problem Total; Competence Total; also Externalizing, Internalizing, Dysregulation, Competence, and Maladaptive Item Clusters	12 to 35 months	20–30 min	600/*** 50 (10)	>0.80 for all scales (>0.90 for Externalizing, Dysregulation) (alpha)	Varies across scales	0.76 to 0.91 (~6 day retest)	Good factor validity; content analysis; convergent and discriminant validity	Discriminative validity for autism versus unaffected (Sensitivity = 100%; Specificity = 89%)	Parent form, parent interview form, and childcare provider form; Spanish translation available; brief screening version (BITSEA)	Little technical information about childcare provider or Spanish forms	[39]
*Organization/Planning*												
Rey Complex Figure Test	Copy Strategy	6 to 89	45 min, including 30 min delayed interval	505 age 6–17; 601 age 18–89/*** R aw & age-corrected	0.94 (inter-rater ICC)		0.92, but retest is problematic concept because of learning	Good evidence of validity for memory	Moderate evidence of discriminant validity	New manual (1996) improves scoring criteria & guidelines, as well as norms. Developmental scoring norms capture problem solving strategy (as opposed to outcome score) which is a key correlate of executive functions that is often not addressed.	Wide developmental variation and limited normative sample compromise sensitivity. Scoring system is complex and prone to error; requires specific training for adequate accuracy.	[40]
*Pragmatic Language*												
Test of Problem Solving -Child and Adolescent (TOPS 3 Elementary)	Pragmatic Language	6 to 12 yr 11 mo	35 minutes	1406/**** 100 (15)	0.56 to 0.69 internal consistency (0.65= average internal consistency across domains); 0.89 inter-rater		0.84	Concurrent and criterion and some discriminative		Assesses language based critical thinking skills	Lengthy to administer.	[41]
*Processing Speed*												
CPT II	Reaction time (Conner's) Omissions d Prime Commissions Variability Standard error	6+	15–20 min	1920^C^/*** 50 (10)	0.95 (split half) 0.94 (split half) 0.83 (split half) 0.83 (split half) 0.66 (split half) 0.87 (split half)	35.02–55.70^B^ 1.16 to 2.66 0.06 to 0.10 10.03– 12.79 0.42 to 0.53 2.60 – 4.59	0.55 0.84 0.76 0.65 0.60 0.65 (Average retest interval of 3 months, N only 23)	Correlations w/CPT and ADHD rating scales range from 0.33 to 0.44 in some studies; CPT overall index and teacher rating correlations were nonsignificant; modest Correlations w/CPT omission errors and teacher ratings; overall mild correlations b/w CPT and rating scales		Standardized task that measures multiple performance facets of attention	Relatively small number of minorities included in the norm sample; overall mild correlations between CPT and ADHD rating scales	[15]
WISC-IV	Processing Speed Index	6 to 16	1–15 min	2200/**** 100 (15)	0.88 (split half)	5.21	.79 (~1 month)	Good	Some evidence of discriminating ADHD from other youths	Strong norms, good reliability	Not validated as stand-alone administration	[20]
*Response Inhibition*												
Behavior Rating Inventory of Executive Functioning (BRIEF)	Inhibit scale	2 to 18 years	10–15 min	1419/**						Parent and teacher forms; inexpensive; collateral source of information about executive functioning. Comprehensive coverage of subdomains of executive functioning; ecologically valid measure; used extensively in research with good sensitivity; easy to administer and complete.	Parent rating are susceptible to bias; report of everyday executive function does not necessarily accurately parse subdomains of executive function. Normative sample not nationally representative; variable correlations between scores and underlying processes	[16]
CPT II	Errors of Commission	6+ years	15–20 min	1920^C^ ***						Standardized task that measures multiple performance facets of attention	Relatively small number of minorities included in the norm sample; overall mild correlations between CPT and ADHD rating scales	[15]
*Social Cognition*												
Social Responsiveness Scale (SRS)	SRS Total Total - Parents (Female) Total - Parents (Male) Total - Teachers (Male) Total - Teachers (Female) Clinical Ratings (Both)		15 min	1636/*** 50 (10)	0.94 (alpha) 0.93 (alpha) 0.97 (alpha) 0.96 (alpha) 0.97 (alpha)	2.4 2.6 1.7 2.0 2.1	0.85 (~17 month) 0.77 (~17 month)	Good -- Discriminative validity (AUC = 0.85 PDD+Autistic vs. psychiatric control and normal)	Diagnostic and longitudinal	Exceptional evidence of construct validity; inexpensive to administer	Norms not fully nationally representative	[42]
*Visual Motor*												
Beery VMI (5th Ed.)		2 to 18 years for full form; 2 to 7 years for short form	10–15 minutes	2512/**** (11,000 over 5 standardization; 2512 in the 2003 norm sample) 100 (15)	0.82 (alpha) and 0.88 (odd-even); 0.92 for inter-scorer ratings of 100	Listed by age ranging from 4 to 6 (5.25 but this is not weighted for number in each group, and the numbers in table were already rounded).	0.89 for 10 day test-retest on 115 kids	Correlates 0.62 w WISC-R, 0.63 w/Comp Test of Basic Skills, 0.89 w/age, 0.52 w/Wide Range Assessment of Visual Motor Abilities, and 0.75 with Developmental Test of Visual Perception-2	Good	Culture free, easy to administer, used in many countries	Scoring somewhat difficult	[43]
*Visual Spatial*												
WASI	Performance IQ	6 to 89	15 min for 2 scales	2245/**** 100 (15)	0.94 (split half)	3.75	0.88 ~1 month	Good	Good, based on WAIS	Brief, excellent precision; validated as brief instrument	No additional constructs covered	[22]
*Working Memory*												
WISC R	Arithmetic	6 to 16	5–7 min	2200/**** 10 (3)	Moderate	Moderate	Moderate	Poor – task combines multiple functions in addition to working memory	Poor	At time, most widely used test	Arithmetic blends multiple neurocognitive functions into single test; WISC-R now outdated	[34]
WISC-IV	Working Memory Index	6 to 16	15–20 min	2200/**** 100 (15)	0.92 (split half)	4.27	0.85 (~1 month)	Good	Good	Measured as factor; strong norms; widely used test	Working Memory tasks not designed to be administered as stand-alone	[20]

A: The manual reports five different “standardization” samples: 1st—453 normal kids southeast urban public school ages 6.5 yr to 17 yr 11 mo; 2nd—49 18 year olds; 3rd—15–77 in TX & CO as control subjects in pesticide poisoning study; 4th -- 50 in CO ages 58-84; 5th -- 124 airline pilots in CO and Washington; 6th -- 73 healthy adults from retirement community in Detroit)

B: The technical manuals do not report a mean or median; numbers presented separately for ages 6 to 17 years. For CPTII, no means or medians were reported for standard error of measurement; SEM given as ranges for ages 6 to 17 years.

C: 1920 non-clinical sample; 378 ADHD cases: 223 adults w/neurological impairment.
